# A Self-Organizing Interaction and Synchronization Method between a Wearable Device and Mobile Robot

**DOI:** 10.3390/s16060842

**Published:** 2016-06-08

**Authors:** Min Su Kim, Jae Geun Lee, Soon Ju Kang

**Affiliations:** 1 Departement of Software Convergence, Kyungpook National University, 80 Daehakro, Bukgu, Daegu 702-701, Korea; totoro0143@gmail.com; 2 School of Electronics Engineering, College of IT Engineering, Kyungpook National University, 80 Daehakro, Bukgu, Daegu 702-701, Korea; worms035@naver.com

**Keywords:** mobile robot, human following or leading, LF/RF pairing, device-to-device synchronization

## Abstract

In the near future, we can expect to see robots naturally following or going ahead of humans, similar to pet behavior. We call this type of robots “Pet-Bot”. To implement this function in a robot, in this paper we introduce a self-organizing interaction and synchronization method between wearable devices and Pet-Bots. First, the Pet-Bot opportunistically identifies its owner without any human intervention, which means that the robot self-identifies the owner’s approach on its own. Second, Pet-Bot’s activity is synchronized with the owner’s behavior. Lastly, the robot frequently encounters uncertain situations (e.g., when the robot goes ahead of the owner but meets a situation where it cannot make a decision, or the owner wants to stop the Pet-Bot synchronization mode to relax). In this case, we have adopted a gesture recognition function that uses a 3-D accelerometer in the wearable device. In order to achieve the interaction and synchronization in real-time, we use two wireless communication protocols: 125 kHz low-frequency (LF) and 2.4 GHz Bluetooth low energy (BLE). We conducted experiments using a prototype Pet-Bot and wearable devices to verify their motion recognition of and synchronization with humans in real-time. The results showed a guaranteed level of accuracy of at least 94%. A trajectory test was also performed to demonstrate the robot’s control performance when following or leading a human in real-time.

## 1. Introduction

**Figure 1:**
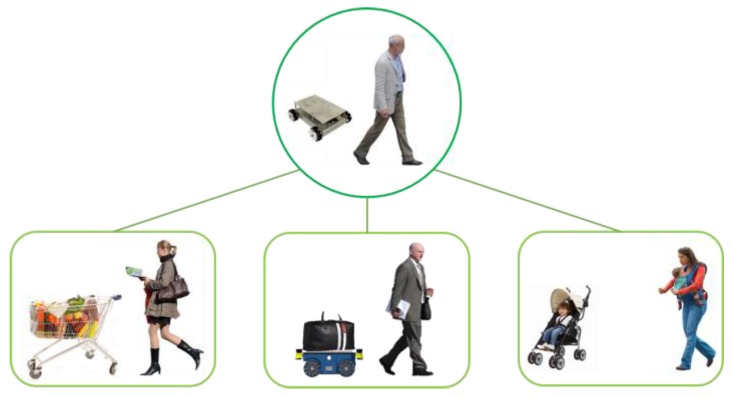
Application of the Pet-Bot system.

The demand and supply of human-support robots continue to increase. However, most of these robots require specific control interfaces. This means that the user can hardly do anything except control when using the robot. If the user could do something else, his/her time efficiency would increase. Human-support robots that can also follow their users mainly use image-processing or sensor network methods to eliminate specific control interfaces. Image-processing methods basically require high levels of hardware specifications. In addition, these methods only perform well in locations with low population densities. In contrast, in locations with high population densities, these methods require additional complex algorithms to maintain the performance which the robots in location with low population density have. Sensor network methods perform well in limited space. These methods have spatial constraints because a specific infrastructure must be constructed. Therefore, we propose a mobile robot system that can accompany a user and does not require a specific control interface through self-organizing interaction from the combination of a wearable device to obtain the user’s motion information and a mobile robot for carriage service. When a robot goes ahead or follows a human, it can also synchronize with the human’s motion by using self-organizing interaction without any specific controls. In case of exceptions, this system requires specific controls that are commanded by a gesture recognition function in the wearable device. The proposed robot is called “Pet-Bot” because, like a pet, it can recognize its master and their location and motion to accompany them. In this system, a Pet-Bot identifies the authorized user and their location by using low-frequency (LF) wireless communication, and the wearable device identifies the user’s motion with the inertial measurement unit (IMU). By using such information, Pet-Bots can always identify an authorized user, go ahead or follow the user, and synchronize with the user’s motion without spatial constraint. The robot can guarantee safe-carriage service when going ahead of the user because the user can easily check their goods in their line of sight. In addition to the areas shown in Figure 1, this robot can be applied in any area that needs carriage services.

The rest of this paper is organized as follows: Section 2 presents an overview of related works. Section 3 describes the detailed design of our work. Section 4 presents the results of experiments using our prototype robot and wearable devices. Finally, Section 5 summarizes our contributions and discusses future extensions.

## 2. Related Research

Two conventional human-following mobile robots have been developed, each of which has its own method for recognizing and following users. One uses image processing, and the other uses a sensor network.

An image-processing method [1,2,3,4,5] uses a different type or number of cameras and algorithms for obtaining the user’s location and motion information. By analyzing images, it identifies the human’s location and steers itself after the human. However, such methods commonly require powerful computing resources including GPUs, as well as high-capacity batteries for long-term operation. These methods essentially incur delays during the image analysis and hence are not suitable for an embedded system requiring a long operation time with real-time interaction.

The next type of method uses a sensor-network [6,7]. This method obtains the user and robot location information using fixed sensor nodes, e.g., a charge coupled device (CCD) camera, laser range scanner, or Radio Frequency Identification Reader (RFID), within a limited space. Because fixed sensor nodes that communicate with each other are applied, the user and robot must be within a limited space; this method can create an absolute coordinate system, and the robot can obtain the absolute coordinates of both the user and the robot, which are needed to follow the user. This method can provide the robot accurate coordinates of the user and itself, but it requires a specific infrastructure and has certain spatial constraints.

In addition, other methods for human-following or guiding robots have been developed. One uses compressed infrared sensors [8] to detect and follow a human. It does not need a powerful processor but has a weak point in terms of its operation in bright locations. Another method uses laser range finder (LRF) sensors to detect the user and guide them through a safe path [9]. This system partially solves some of the problems described above. However, it cannot synchronize with the user’s motion.

To identify a human’s location, some methods might require a Pedestrian Dead-Reckoning (PDR) system. Inertial Navigation Systems (INSs) [10,11,12] and Step-and-Heading Systems (SHSs) [13,14,15] are two major topics of a PDR. An INS uses an Inertial Measurement Unit (IMU) that attaches to the human body, e.g., the head, waist, or toe. The INS filters the data from the IMU and then uses additional compensation functions to obtain the user’s velocity and direction. The SHS uses ultrasonic sensors as well as an IMU attached to the human’s body. The SHS filters the data from the sensors and then estimates the user’s steps and direction. To increase the accuracy, both methods use filters and compensation functions, which have a long processing time.

To solve the above constraints, an LF wireless communication method [16] is proposed in this paper. Because this method simply uses an LF signal’s received signal strength indication (RSSI) to obtain the human’s location, it does not require a powerful processor, complex algorithms, or a high-capacity battery. It also does not need a PDR system, which may generate certain timing issues. In addition, because it uses relative coordinates, it does not incur a spatial constraint. Through a smart belt with an IMU, it can detect and synchronize with a human’s motions. In addition, it uses an LF signal’s unique wake-up patterns to identify the robot’s master. In brief, it can identify the master and their location and motion in real-time using simple algorithms that utilize LF wireless communication and an IMU.

## 3. Detailed Design

### 3.1. Basic Concept

**Figure 2:**
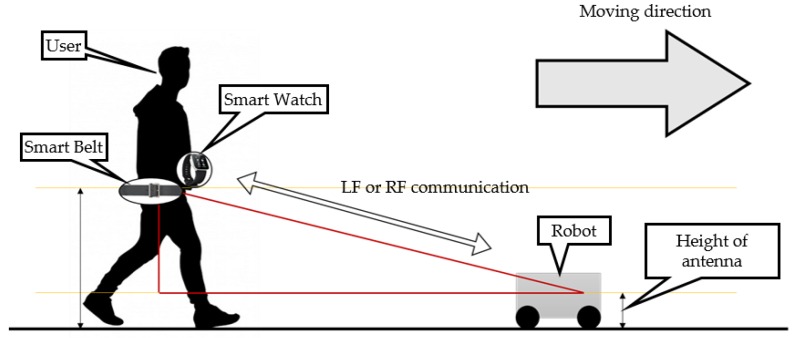
Overall setup of the Pet-Bot system.

Figure 2 shows the overall setup of the Pet-Bot system. It consists of wearable devices, a smart watch and belt, and a mobile robot. If a user wearing a smart watch and belt approaches the mobile robot, an LF signal having a unique wake-up pattern is periodically burst from the robot, and if the user is authorized, the user’s smart belt will wake up and then connect with the robot using Bluetooth low energy (BLE) communication and then services to the user are offered. If the user selects a leading mode, the mobile robot finds the user’s location and motion information from the smart belt. The user’s location is detected using the LF signal’s RSSI, and the motion is recognized through the IMU in the smart belt. Finally, using the information regarding the user’s location and motion, the robot can accompany the user.

The smart watch in this system is used to give commands whenever the robot requires it, exception situation and remote-control mode. When the robot detects an obstacle in front of the robot, in leading mode, an exception situation occurs. In both cases, the user has to provide the direction where the robot is to move toward. To command the robot, we use a smart watch gesture recognition function. Using this function, the user is able to command the robot in a particular direction in a very intuitive way because the user simply points toward the direction where the robot is to move. In this paper, we use the seven basic gesture patterns shown in Table 1. Through a combination of gestures, the robot can even move in a diagonal direction. The principle of this function is based on analyzing the pattern of accelerometer values.

**Figure 3:**
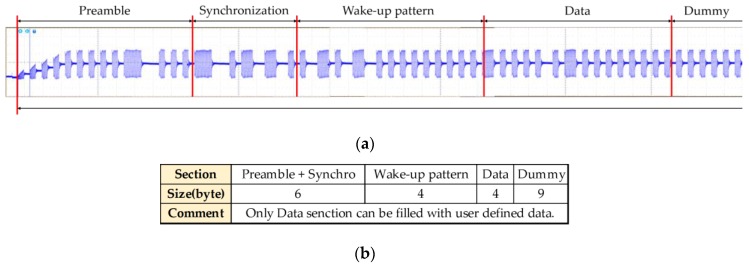
Protocol of LF wireless communication in Pet-Bot system. (**a**) LF signal wave; (**b**) message format for LF wireless communication.

Because a low-frequency band has high transmissivity, such a signal can transmit through obstacles. Thus, an LF signal can be used for obtaining the distance and direction from the object. This technic is widely used in smart key systems for vehicles. Figure 3 shows the protocol of the LF wireless communication used in the Pet Bot system. As can be seen, there are only 4-byte data sections that can be filled with user-defined data. Such sections are insufficient to contain all of the user’s location and motion information. Moreover, the robot has only two LF transmit antennas, and wearable devices include only LF receivers. Therefore, we additionally use RF BLE wireless communication to exchange the needed data.

**Figure 4:**
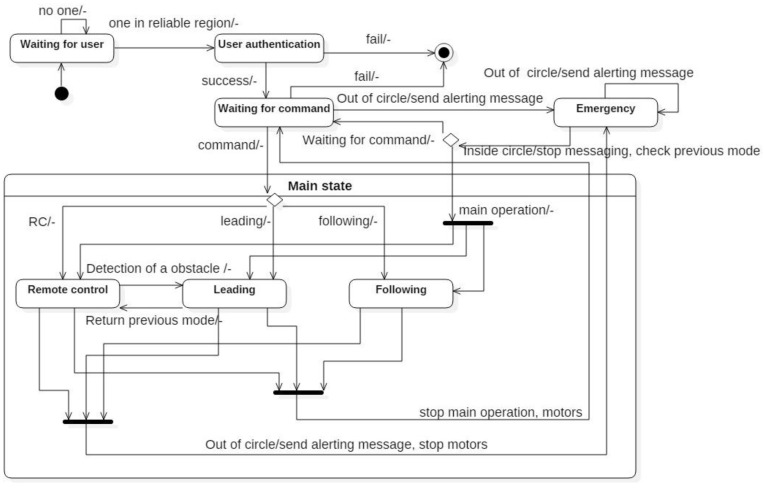
State diagram of a mobile robot.

Figure 4 shows a state diagram of a mobile robot. When the robot is first turned on, it enters into a waiting-user sequence and sends out an LF signal periodically. When a user wearing an authorized smart belt is within 1 m of the robot, the robot identifies whether the user is its master. If the user is its master, it enters a command-waiting state. In this state, the user can select the main operation: remote-control mode, leading mode, or following mode. If the main operation is stopped, the robot returns to a command-waiting state. If the robot is far from the user, it enters into an emergency state. In this type of situation, the robot sends an alert message to the smart belt. In leading mode, when the robot detects any obstacles to the front, the robot will change to remote-control mode. After avoiding the obstacle, if the robot receives a return command, it will change back to the previous mode.

### 3.2. Detection of its Master

**Figure 5:**
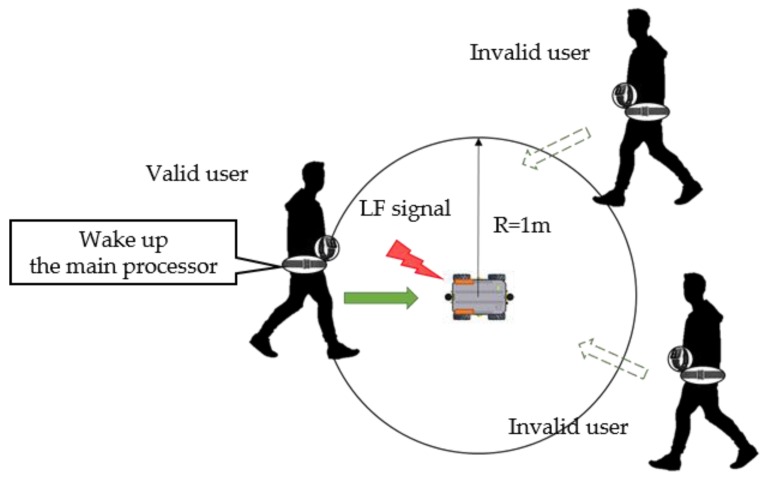
Master recognition.

Figure 5 shows the detection of the robot’s master. Once the mobile robot is turned on, it sends LF signals every 3 s. If the user is within 1 m of the robot, their smart belt can receive the robot’s LF signal. The LF signal has its own wake-up pattern, and the LF receiver maintains this wake-up pattern in its register. Therefore, when the smart belt receives an LF signal, it compares the received pattern with its own pattern in the register. After confirming the pattern, it generates an interrupt to wake up the main processor. It then sends a reply to receive services. Through these LF signals, the mobile robot can identify its master [16]. In addition, smart watch authorization is also checked in the same manner when needed.

### 3.3. User-Location Identification

**Figure 6:**
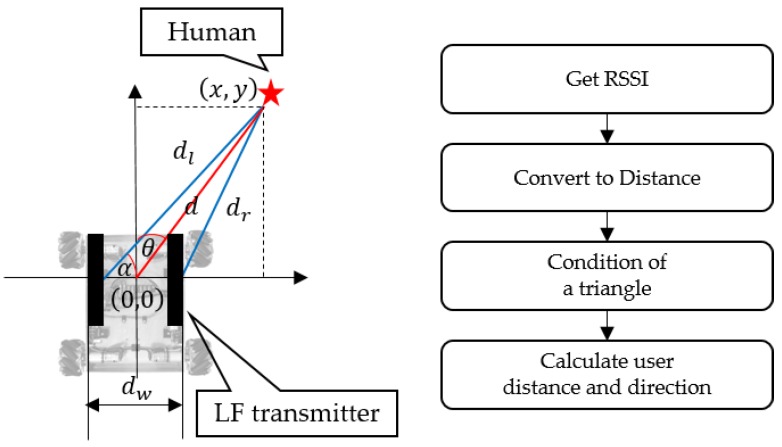
Method for obtaining a user’s location information.

To accompany a human, the mobile robot must know the user’s location. The user’s location information is available from the LF signal’s RSSI [17]. In other words, the mobile robot can calculate the user’s distance and direction using the LF signal. To identify the user’s location, it is first necessary to make a table that maps the RSSI signal strength to the distance. Once the mobile robot begins the leading or following services, it sends LF signals every 350 ms. As soon as the user’s smart belt receives an LF signal, it obtains the RSSI values from the LF receiver and sends the values to the mobile robot through RF BLE wireless communication. Figure 6 shows how the user’s location information is obtained.

The mobile robot is shown on the left side of Figure 6, and the right side describes the sequence used to obtain the user’s location information. Two LF transmitter antennas are placed vertically side-by-side, 20 cm apart. They each send LF signals in turn; the mobile robot can then obtain the RSSI value from the user’s smart belt through RF communication.

We created an RSSI value table using the distance, and thus we can determine the distance from the RSSI value. The robot then obtains the distance to the left antenna ($d_{l}$) and right antenna ($d_{r}$). Through these two distances and distance between two antennas ($d_{W}$), a triangle is formed. By determining the condition of the triangle, we judge whether the triangle is formed:

(1) $$d_{B}+d_{C}>d_{A}\;\left(d_{A}\;is\;the\;longest\;side\right)$$

The coordinates *(x, y)* of the user’s location are then calculated for use by the mobile robot’s relative coordinate system by utilizing a trigonometric function. Equations (2) and (3) indicate how the user’s location is calculated. Here, α is the angle contained between sides of length $d_{l}$ and $d_{r}$, *X* and *Y* are the coordinates of the user’s location, *d* is the distance between the robot and the user, and finally, *θ* is the direction of the user, which represents the angle away from the current direction:

(2) $$\{\begin{array}{c} \alpha =\cos^{-1}\left(\frac{d_{l}^{2}+d_{w}^{2}-d_{r}^{2}}{2d_{l}d_{w}}\right)\\ x=\;d_{l}\cos\alpha -\frac{d_{W}}{2}\\ y=\;d_{l}\sin\alpha \end{array}$$

(3) $$\{\begin{array}{c} d=\sqrt{x^{2}+y^{2}}\\ \theta =\tan^{-1}\left(\frac{x}{y}\right) \end{array}$$

**Figure 7:**
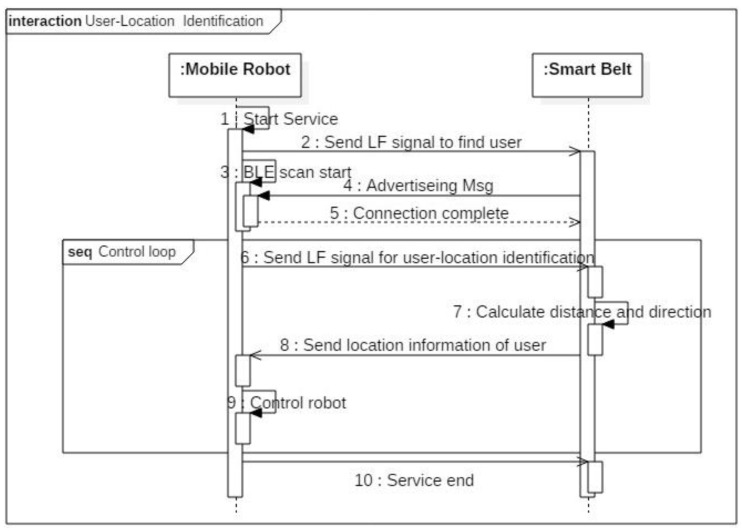
Sequence diagram for finding the user’s location.

Figure 7 shows the sequence diagram for finding the user’s location. The system includes two parts: the mobile robot and a smart belt. Once the mobile robot starts operating, it finds its own master. If the master is within 1 m of the robot, the LF receiver in the smart belt can receive the LF signals. If LF signal’s wake-up pattern is identical to smart belt’s, main processor wakes up and it carries out a BLE connection to provide service. The user can then select the main operation: leading, following, remote control, or other service. If the robot is in leading mode, it sends LF signals periodically to identify the human’s location. As soon as the smart belt receives LF signals from the two antennas, it calculates the distance based on the RSSI value and sends the distance to the mobile robot. Finally, the mobile robot adjusts itself to accompany its master through the user-location information.

The user’s location is reliable when the user is within 2 m of the robot; if the distance between them is greater than 2 m, the LF signal becomes too weak and the RSSI value cannot be calculated. Therefore, it is necessary to consistently synchronize with the human in order to avoid missing them after a sudden movement.

### 3.4. User-Motion Recognition

The robot can recognize two states of user motion: movement and rotation. A movement state occurs when the human is standing, walking, or running. A rotation state relays information on the rotation, angular speed, and accumulated angle of the human. During a movement state, the mobile robot must trace the human even if the human is running quickly or suddenly stops. Otherwise, it may lose track of, or even run into, the human.

**Figure 8:**
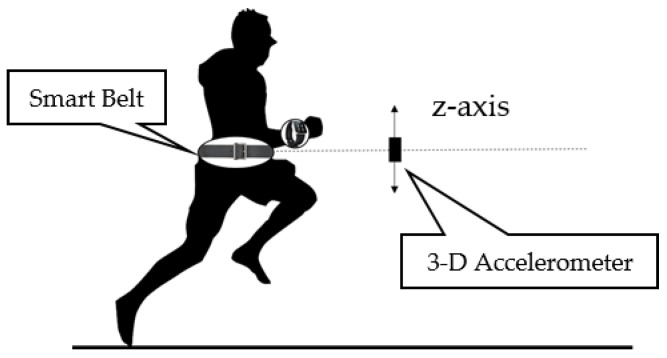
Accelerometer shaking when the user is running.

Figure 8 shows how the user’s movement state is recognized through the accelerometer. We use the smart belt’s accelerometer attached to the user’s waist to determine their state of movement. As shown in Figure 8, when the human moves, an oscillation occurs on the z-axis, which causes a wave in the accelerometer. In Table 2, we classify the user’s movements by using the amplitude of the accelerometer value, as well as its periodicity. The periodicity is defined by checking the number of sign changes of the values that occur within a valid time period. If the periodicity indicates that the user does a periodic action, then the smart belt applies the signal magnitude area (SMA). The SMA accumulates the accelerometer values within the programmer-defined time. It can be used to classify a user’s movements. Table 2 shows the classification rules for user movements based on the periodicity and SMA. Through this process, we can classify a user’s movements accurately.

**Figure 9:**
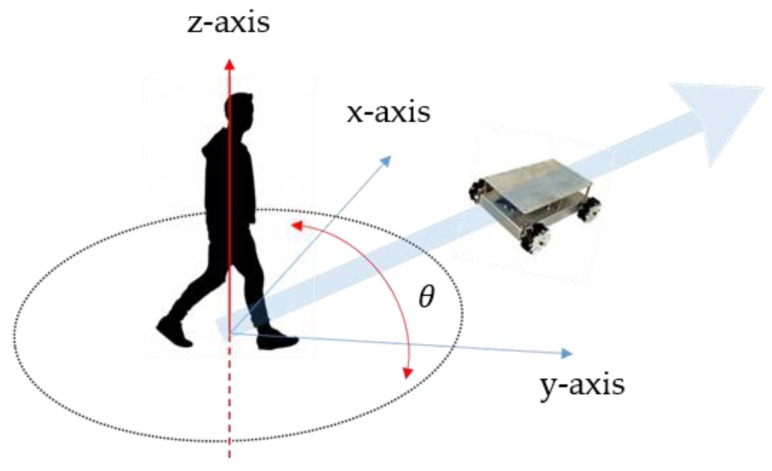
Detection of user rotation.

The next motion state is a rotation. When leading the user, the mobile robot must be aware of the human’s rotation information, because it must mimic the user’s direction when the user turns. Figure 9 shows the user-rotation recognition for a leading task. If the user turns by *θ*, the mobile robot must correct its movement direction. Because the robot uses Mecanum wheels [18], it is not necessary for it to rotate and move; it can simply move along the arc of a circle.

The user rotation information is found through the gyro sensor in the smart belt. The gyro sensor value is sampled at 70 ms intervals. It minimizes the amount of noise by using a moving-average filter. The robot must know the rotation state, angular speed, and accumulated angle of the human to synchronize with their rotation. Using this information, the mobile robot can recognize the human’s rotation and accompany the human in a leading mode.

### 3.5. Control System for Leading or Following a Human

**Figure 10:**
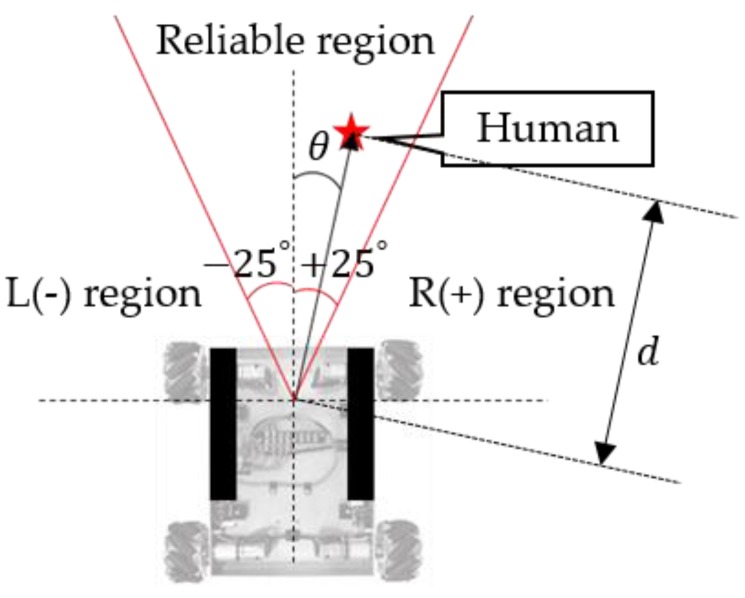
Control region of the mobile robot.

**Figure 11:**
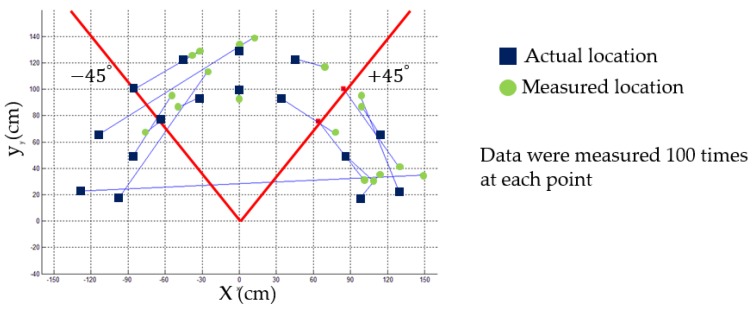
Experiment result in searching for a reliable region.

When leading or following the human, the goal of the robot control is to maintain a specific distance and direction from the user. To do this, the user location and motion information are required, which are attainable from the smart belt and LF wireless communication. Figure 10 shows our control region. The mobile robot has a different directional-angle resolution depending on how the antennas are located. Thus, it has a ±25° “reliable region.” In other words, the robot must maintain a specific distance (1 m), and stay within this reliable region. We set up this reliable region based on our experimental results. As shown in Figure 11, distortion of the LF signal worsens outside of this region.

**Figure 12:**
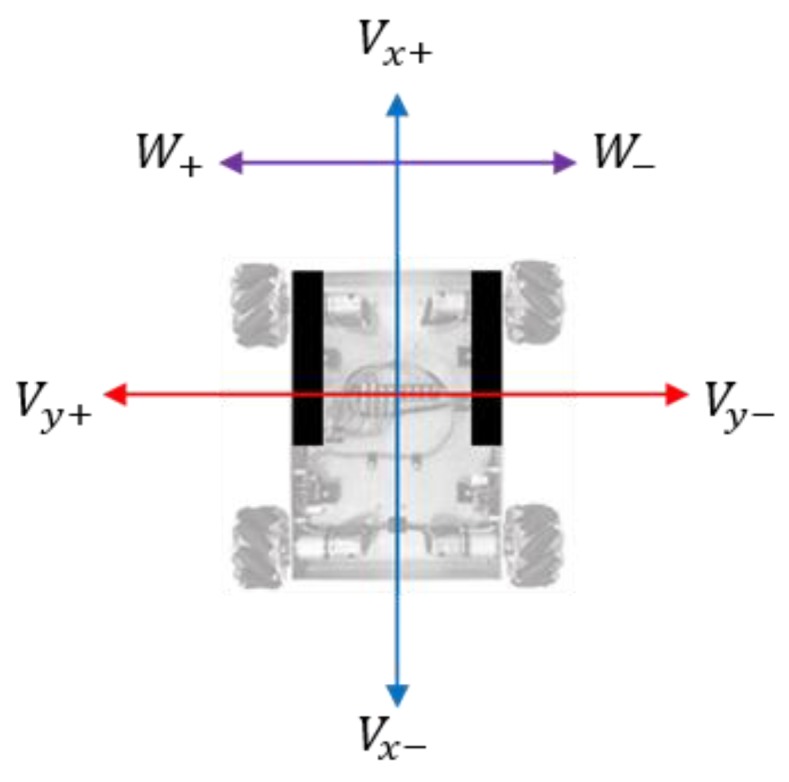
Control factors of the mobile robot.

Three factors control the robot: *Vx*, front and rear speed; *Vy*, left and right speed; and *W*, angular speed. The robot uses Mecanum wheels, which when combined with the speed factors above, allow the robot to move quite freely without having to rotate. Figure 12 describes the control factors and their signs.

**Figure 13:**
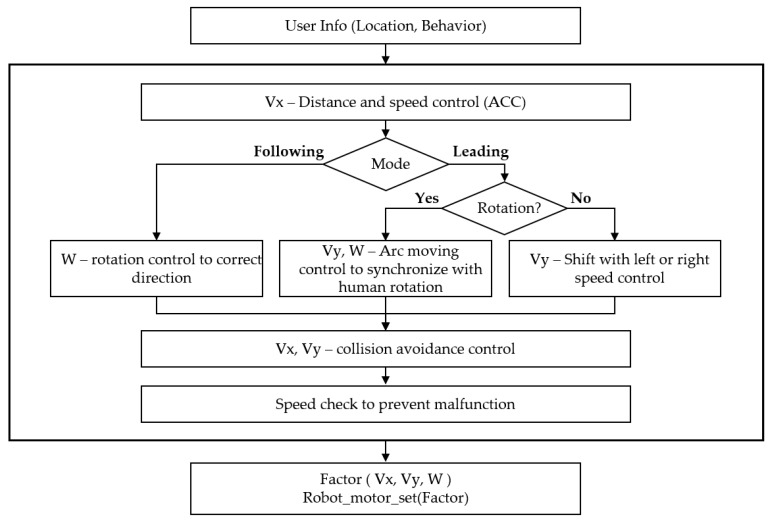
Control system of a mobile robot when following or leading a human.

Figure 13 shows the control system of the mobile robot. It can obtain the user’s location and motion information from the smart belt. This user information is the input of the control system. The first controller is the distance and speed controller. To maintain a specific distance (1 m), it uses automobile adaptive cruise control (ACC) [19]. When the distance is far from the target distance, it operates as a distance controller. However, when it is near the target distance, it controls the speed to keep pace with the human.

The next controller varies with the operation mode. In following mode, it uses the rotation controllers to correct the direction, e.g., if the user is in the right-side region, it turns to the right to keep the user within the reliable region. In leading mode, on the other hand, the controller depends on the user’s rotation state. If the user is rotating, the robot synchronizes with the human rotation by utilizing a moving arc. If the user is not rotating, and the direction region is not within the reliable region, the robot moves left or right to correct its direction. The final step is to avoid walls or obstacles, and check its speed to prevent a malfunction. The output of this control system is a control factor, *Vx*, *Vy*, or *W*. The robot operates its motor according to these control factors.

## 4. Implementation and Evaluation

### 4.1. Mobile Robot Hardware

**Figure 14:**
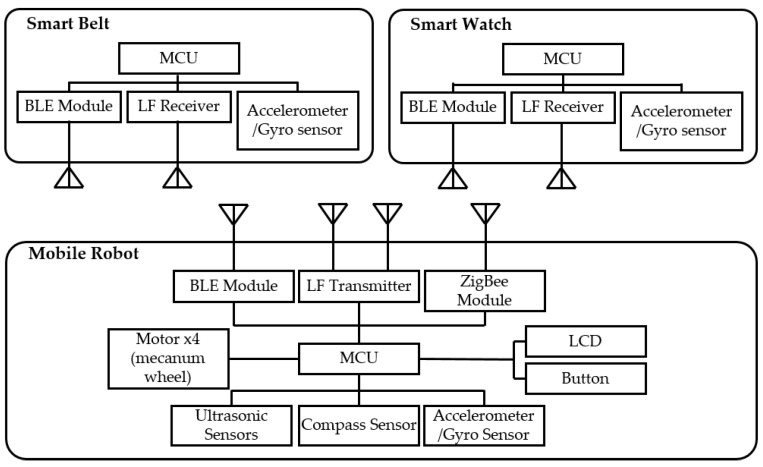
Hardware configuration of the mobile robot system.

Figure 14 shows the hardware configuration of the mobile robot system. There are two parts: the wearable devices (smart watch and belt) and the mobile robot. The wearable devices consist of an ARM Cortex-M3 based MCU (1024 KB flash, 128 KB RAM), LF receiver (low-power LF wake-up), a BLE module, and an IMU (three-axis independent acceleration and angular rate channels).

**Figure 15:**
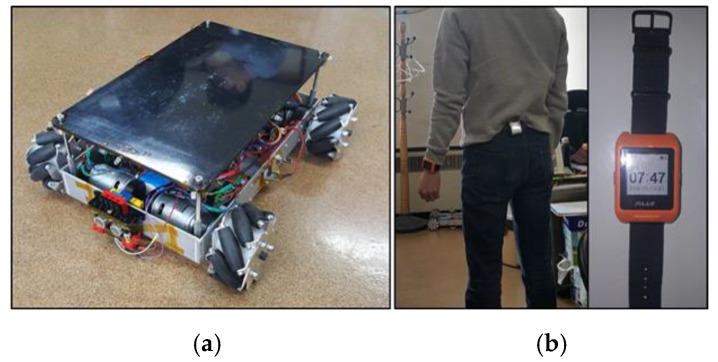
Prototypes of a mobile robot system: (**a**) mobile robot and (**b**) wearable devices (smart belt and watch).

In addition, because wearable devices have a low capacity battery, the components used in the wearable devices basically support low power consumption. The mobile robot consists of an ARM Cortex-M4 based ESTK board, which is a development kit made by our laboratory (1024 KB flash, 128 KB SRAM, fast floating-point calculation), two LF transmitter antennas (with a long reading distance), RF module (low power consumption), four motors with Mecanum wheels, ultrasonic sensors, and a gyro sensor. Figure 15 shows the prototypes of the mobile robot and wearable device used. A performance test was executed using these prototypes. The user wore a smart watch and belt. We conducted three tests for the evaluation: location identification, motion recognition, and trajectory. The first two tests obtained user information. The last test was conducted to observe the mobile robot’s following or leading capabilities using the control system.

### 4.2. User-Location Identification Test

**Figure 16:**
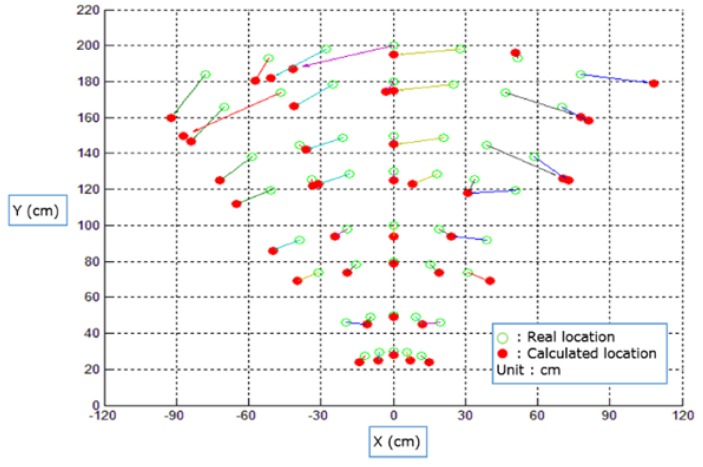
Location identification test result, with varying distances from the user.

Figure 16 shows the results of the location-identification test. The empty green circle is the actual human location. The colored red circle is the measured location. The location is measured while varying the distance between the LF transmitters and the LF receiver from 25 cm to 200 cm. We measured the RSSI values of the LF signal 50 times at each point. Table 3 shows that the results were a 5.10-cm root mean square error (RMSE) and 6.52° RMSE.

### 4.3. User-Motion Recognition Test

**Figure 17:**
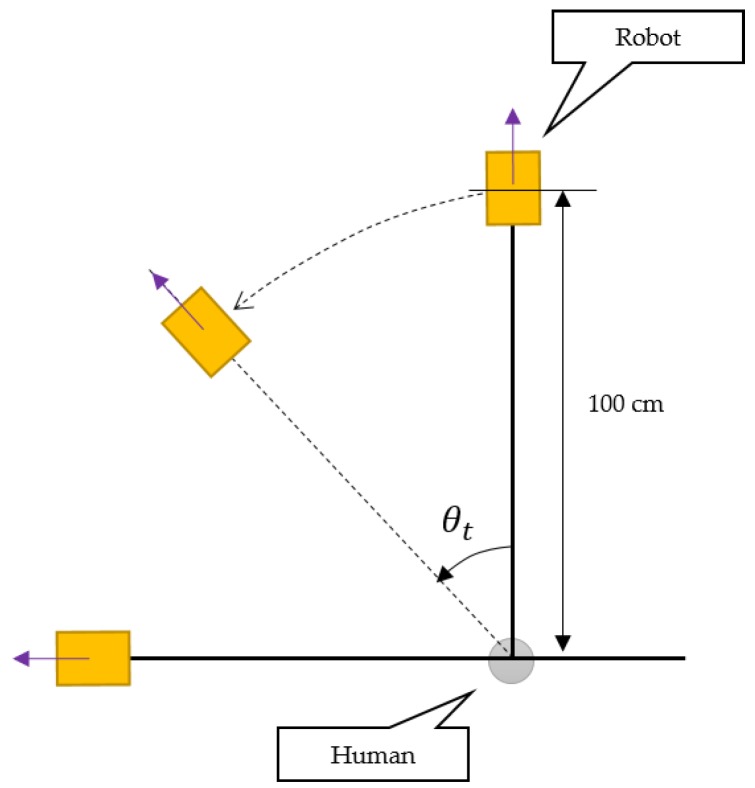
Test environment for user-motion recognition and synchronization.

Figure 17 shows the setup for the user-motion recognition test. The robot is in leading mode, and the distance between the robot and human is 100 cm; the angular speed is fixed. The goal of this test is to verify the smart belt’s rotation-recognition capability and synchronization performance. Therefore, the human rotates at a given angle (−90°, −45°, 45°, and 90°), and we measure the resulting angle of the robot’s arc movement. Table 4 shows the results of this test. Each case has a standard deviation of within 4.0° and a level of precision of over 94%, showing that the robot can be accurately controlled.

### 4.4. Trajectory Test

As mentioned earlier, the mobile robot is controlled through the user location and motion information. To control the robot effectively, an independent control system was implemented. Therefore, we conducted four trajectory tests to see if the robot can be controlled stably. In the following figures, the user is marked as a gray arrow or light-blue circle. The mobile robot for a leading task is marked as a red square. For a following task, the robot is shown as a green triangle.

**Figure 18:**
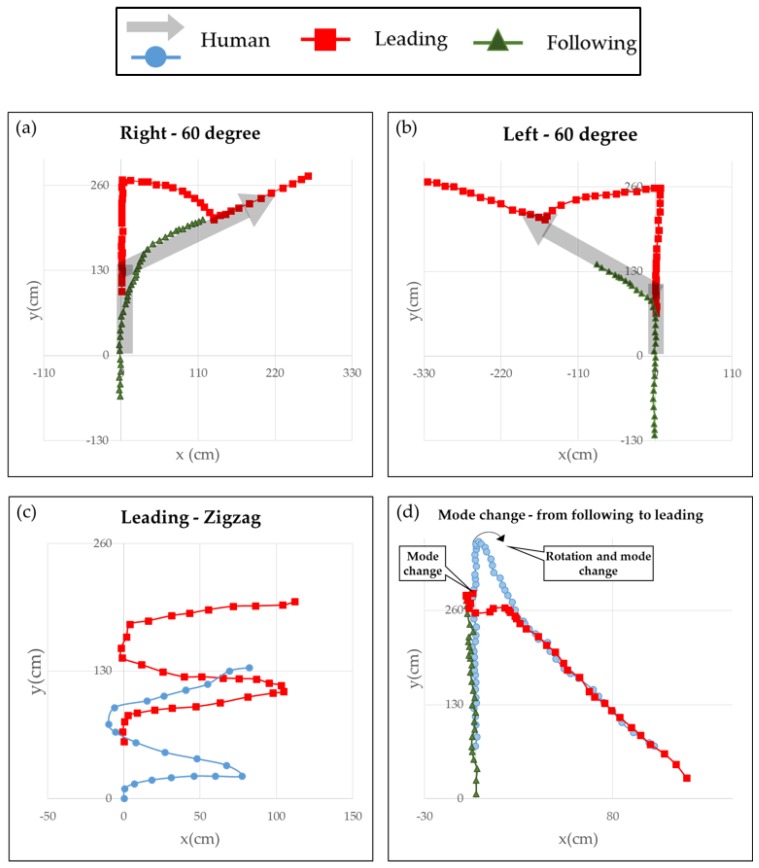
Results of the trajectory test: (**a**) turning right at 60°; (**b**) turning left at 60°; (**c**) zigzag; and (**d**) mode change.

The first two cases tested the control of the straight and rotating movements. The user moved straight, turned to the right or left at 60°, and continued in that direction. The tests were carried out using each driving mode. We can see in Figure 18a,b that the robot is controlled. For the leading task, when the user turned, the robot followed a moving arc, whereas for a following task, it simply rotated at a misaligned angle. Figure 18c shows the performance of lateral movement for a leading task. Figure 18d shows the results of the mode-change test. The user started with the robot in following mode and walked straight ahead; the user then switched the robot to leading mode by turning at a particular point and walking in the opposite direction. As shown in the figures, the system performed well by allowing all major functions to interact with each other, *i.e*., identification of user’s location and motion, and independent control of the robot. And you can see a demo video at [20].

## 5. Conclusions

In this paper, we have proposed a mobile robot system, called Pet-Bot that can accompany a user and does not require a specific control interface through self-organizing interaction from the combination of a wearable device to obtain the user’s motion information. To identify an authorized user and their location by the Pet-Bot itself, we use LF wireless communication. The wake-up pattern of the LF protocol is used to identify an authorized user. The authorization time is very fast, compared with image-processing methods, and provides very accurate authorization results. In addition, we simply use the RSSI of an LF signal to obtain the precise proximity information regarding the user’s location, even though we use very low performance wearable devices, so we can obtain the proximity information of the user’s location quickly and accurately, as shown in the evaluation test. In this system, the robot can synchronize with a user’s motion by utilizing the smart belt’s IMU. Therefore, the robot can react to change of the user’s motion without any specific controls; e.g., run, walk, stop and rotating. This system can provide smooth leading or following driving motion. In the evaluation, we observed an identification performance with greater than 94% accuracy. Through a trajectory test, we proved that the Pet-Bot can be effectively controlled. And we observed a synchronization performance with greater than 94% accuracy. The designed control system has enough performance to lead or follow their user smoothly. As a result, we expect the Pet-Bot system to be applicable at any region that requires carriage services. In the future, the Pet-Bot’s reliability against failures that occur from a distorted LF signal must be improved, and a swarm or combination of robots will be needed to provide more advanced carriage services.

## Figures and Tables

**Table 1:** Basic patterns for gesture recognition.

Symbol	Motion	Command Description
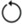	Circle	Cancel (return to command wait mode)
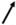	Forward	Move forward
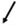	Backward	Move backward
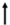	Up	Rotate counter-clockwise 90°
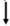	Down	Rotate clockwise 90°
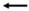	Left	Move left
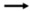	Right	Move right

**Table 2:** Classification rules for user movements.

Periodicity	SMA	State
Movement	3	Run
	2	Walk
	1	Rest
Non-movement	X	Stop

**Table 3:** RMSE of measurement experiment.

X (cm)	Y (cm)	Distance (cm)	Direction (°)
15.57	8.96	5.10	6.52

**Table 4:** Results of user-motion recognition and synchronization test.

Target (°)	−90	−45	45	90
Average (°)	−87.4	−43.8	45.16	87.56
STDEV (°)	3.2	3.6	3.0	3.5
Accuracy (%)	96.04	94.31	94.67	95.78
